# Outbreaks of highly pathogenic porcine reproductive and respiratory syndrome in Jiangxi province, China

**DOI:** 10.1186/2046-0481-65-14

**Published:** 2012-07-11

**Authors:** Aijiang Guo, Guohua Wu, Wei Gong, Xuenong Luo, Haixue Zheng, Huanjie Jia, Xuepeng Cai

**Affiliations:** 1 State Key Laboratory of Veterinary Etiological Biology, National Foot and Mouth Disease Reference Laboratory, Gansu Provincial Engineering and Technique Research Centre on Biological Detection, Lanzhou Veterinary Research Institute, Chinese Academy of Agricultural Sciences, 1 Xujiaping Yanchangbu Chengguan District, Lanzhou, 730046, China

**Keywords:** Highly pathogenic, PRRS, ELISA, RT-PCR, NSP2, Swine herds

## Abstract

In 2007, herds of pigs in Jiangxi Province, China experienced outbreaks of a severe form of suspected porcine reproductive and respiratory syndrome (PRRS) characterized by high fever, high morbidity and mortality in animals of different ages. 152 swine sera and 42 tissues (consisting of liver, lung, lymph node and kidney) from five herds of pigs were collected. Pigs were diagnosed as infected with a highly pathogenic form of the PRRS virus (PRRSV) based on ELISA and reverse transcriptase polymerase chain reaction (RT-PCR) results. Serological surveys indicated that 67-100% of the examined pig herds in Jiangxi Province were seropositive. 42 tissue samples were used to detect classical swine fever virus, porcine circovirus type 2 and PRRSV. Results indicated that only PRRSV was detected in 42 samples. 12 PRRSV amplified products of five herds, which consisted of two or three samples randomly selected from each herd, were used for sequencing. Subsequent nucleotide sequencing showed that the NSP2 gene had 99–99.7% nucleotide and 99.2–100% derived amino acid sequence identities among 12 tissues with that of the PRRS-JXA1 strain, deletions of 29 amino acids corresponded to positions 534–562 of the NSP2 gene sequence. These results revealed that the diseased pigs were all caused by fatal PRRSV variant. Compared with the same period in 2006, the number of positive cases from Jiangxi Province remained unchanged. These findings demonstrated that the highly pathogenic Northern American type PRRSV was still spreading in Jiangxi Province, China in 2007.

## Background

Porcine reproductive and respiratory syndrome (PRRS) is caused by the porcine reproductive and respiratory syndrome virus (PRRSV). This virus was first isolated in 1991 and later identified as a small enveloped virus with a polyadenylated RNA genome, which contains eight open reading frames (ORFs) of approximately 15 kb in length [1,2]. PRRSV is considered to be one of the most economically important pathogens of pigs [3-7]. PRRSV strains have been grouped into European (Lelystad) types and North American (GenBank: VR2332) on the basis of genetic and antigenic differences between the isolates from the two continents [8]. The JXA1 isolate belongs to the North American PRRSV genotype by phylogenetic analysis based on the nucleotide sequence of the structural protein ORFs [9]. Although different PRRSV strains cause similar diseases in pigs, previous studies have shown that the antigenicity and pathogenicity vary substantially among PRRSV strains identified around the world [10-15]. Many studies have shown that the non-structural protein 2 (NSP2)-coding gene (nsp2) in the PRRSV genome may represent the most genetically variable region [16-19].

HB-2 exhibited variations in the NSP2 nonstructural protein with a deletion of 12 amino acids in comparison with other North American PRRSV isolates [5]. A 29 aa deletion in the NSP2 protein of JXA1 occurs from amino acid positions 534 to 562 [9], which could be used as a molecular hallmark. PCR-based assays showed that many molecular markers for PRRSV could be amplified using primers specific for unique gene fragments of PRRSV [20-22].

Here, we report the outbreaks of highly pathogenic PRRSV in five herds of pigs in Jiangxi Province, China. Using the IDEXX PRRS ELISA serology test kit we examined sera, and the RT-PCR technique was used to amplify the NSP2 segments from the samples of deceased pig lung to obtain further molecular evidence.

## Case presentation

### Methods

#### Serum and tissues

No PRRSV vaccination had been carried out on any of the pig herds. Sera and tissue samples of lymph nodes, lungs, livers, and kidneys from diseased pigs suspected of being infected with PRRSV were collected in 2007 from pig herds located in Jiangxi Province, China, where pigs had high morbidity and mortality rates.

The North American PRRSV strain JXA1, isolated in our laboratory, and the attenuated vaccine strain Ch-1a (GenBank: AY032626) saved by our lab were used as positive controls. Pigs that were bred in Laboratory Animal Center of Lanzhou Veterinary Research Institute which did not show any clinical symptoms or gross lesions were used as negative control pigs. All animals were handled in strict accordance with good animal practice according to the Animal Ethics Procedures and Guidelines of the People’s Republic of China, and the study was approved by the Animal Ethics Committee of Lanzhou Veterinary Research Institute, Chinese Academy of Agricultural Sciences (No. LVRIAEC-2010-002).

### Enzyme-linked immunosorbent assay (ELISA)

Antibody titers were determined using a commercially available ELISA kit (IDEXX HerdCheck PRRS, Westbrook, Maine). According to the manufacturer’s instructions, tested serum samples for which the optical density ratio is ≥0.4 were considered to be positive against PRRSV.

### RNA extraction and RT-PCR

42 frozen clinical samples were homogenized for 15–30 s with a homogenizer [23]. Viral genomic RNA was extracted simultaneously from homogenized tissues and from lysates of infected cell cultures (positive control) with the TaKaRa MiniBEST Viral RNA Extraction Kit Ver.3.0 according to the manufacturer’s protocol (TaKaRa, Dalian, Japan). RT-PCR of partial Nsp2 was performed using TaKaRa One Step RT-PCR kit (TaKaRa, Dalian, Japan) following the manufacturer’s instructions. Comparisons of the whole genome sequence of four variants (HeB, HuB, JXA1, and HuB1, GenBank accession numbers EF112447, EF112446, EF112445, and EF075945, respectively), and the Northern American strain VR2332 (GenBank accession number AY150564), sequence of the nucleotide deletion region (30 amino-acid deletion in Nsp2 of highly pathogenic PRRS) and the conserved region in Nsp2 segments were obtained. The nucleotide deletion region was used as molecular hallmark. Specific primers for PRRSV were used to amplify the partial Nsp2 segments. The primer pairs NSP2-F (5’- TGG GCA GCC GAG CAG GTT GAT TTA -3’) and NSP2-R (5’- GTG GGG CGG CGG TGT CTC G -3’) were used to generate an expected 855-bp fragment for the deletion form or a 945-bp fragment for the non-deletion form of PRRSV strains (from nt 2566 to 3420 of the JXA1 strain, GenBank accession number EF112445, and from nt 2567 to 3511 of the CH-1a strain, GenBank accession AY032626). Meanwhile swine fever virus and porcine circovirus type 2 were also detected by RT-PCR and PCR Technique according published methods [24,25].

### Nucleotide sequencing

12 amplified products of five herds, which consisted of two or three samples randomly selected from each herd, were purified using a PCR purification kit (Qiagen, Germany) and cloned using the pGEM-T easy Vector System I (Promega, USA) according to the manufacturer’s instructions. The target fragments were sequenced using a Big Dye Terminator sequencing kit on an ABI 3730 DNA sequencer (Applied Biosystems, USA).

## Results and discussion

### Clinical signs

**Figure 1:**
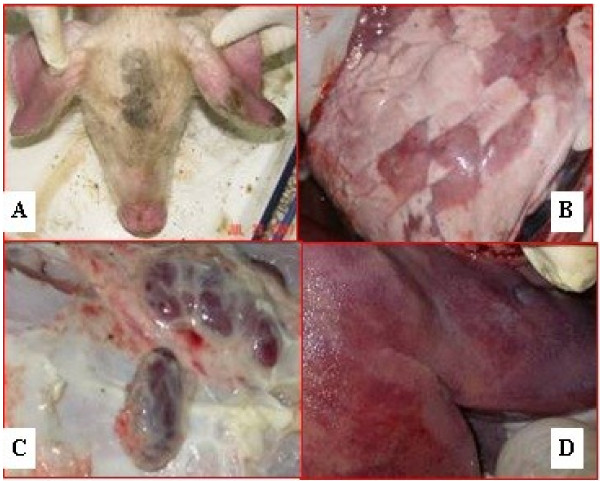
Clinical signs and pathological changes in pigs caused by PRRSV.

In the five affected swine herds, disease was characterized by a prolonged high fever of above 41°C, inappetence and clear lethargy, followed by the development of respiratory distress, nervous signs, red discoloration in skin, and cyanopathy in the ears (Figure 1A). Lactating sows and piglets fell ill first. The main clinical signs in lactating sows were fever, anorexia, decrease of milk yield and nasal discharge. Few lactating sows had nervous signs. Piglets mainly exhibited high and continuous fever (over 41°C), and nervous signs. Weanling and grower pigs became sick later and the clinical signs included continuous fever. The body and ears, and intermittent nervous signs. Nervous signs included incoordination and the adoption of unusual stances, which quickly progressed to opisthotonus and an inability to stand After 10–15 days, the disease quickly spread throughout each herd, lasting one month. The average mortality rates recorded on the five farms was 51% in boars, 72% in sows, and more than 85% in suckling piglets and nursery pigs (Table 1).

**Table 1:** Mortality in pig herds caused by PRRSV according to age and sex

**Herds**	**Dead no./total no.**		
	**Boars**	**Sows**	**Suckling piglets and** **nursery pigs**
1	4/8	36/48	34/36
2	3/7	22/33	32/38
3	6/11	51/67	44/51
4	2/5	14/18	28/34
5	4/6	29/36	17/22
Mean	19/37 = 51%	152/202 = 72%	155/181 = 85.6%

### Pathological changes

The diseased pigs which displayed clinical signs, as mentioned above, were subjected to necropsy examinations. The common lesions included interstitial pneumonia (Figure 1B) associated with hemorrhage and fluid exudation, swollen haemorrhagic lymph nodes (Figure 1C), bronchial and hemorrhage, large number of hemorragic areas in kidney and liver (Figure 1D), intestine ulceration, gastric hemorrhage as well as meneaingeal hyperaemia hemorrhage in meninges. The lymph follicles, spleens, and kidneys had severe histopathologic changes.

### Screening of swine sera by ELISA

Serological tests indicated that 67-100% of these pig herds in Jiangxi Province were PRRSV seropositive (Table 2).

**Table 2:** Seroprevalence of antibodies against PRRSV in pigs in Jiangxi Province, China in 2007.

**Herds**	**No. of positive sera**	**No. of sera examined**
1	10	10
2	10	10
3	14	16
4	17	24
5	62	92
Total	113	152

### Detection of classical swine fever virus, porcine circovirus type 2 and PRRSV

In this study, positive controls and 42 tissues from five pig herds experiencing severe disease outbreaks in 2007 were all positive for PRRSV, and negative for classical swine fever virus and porcine circovirus type 2. Control pigs did not show any clinical signs or gross lesions, and lungs were negative for PRRSV by RT-PCR. Subsequent sequencing analysis of 12 samples revealed a 29-aa deletion corresponding to positions 534–562 of Nsp2. They shared 99.2–100% identity in the predicted amino acid sequence with strain JXA1 isolated in 2006, which had deletions of one and 29 amino acids relative to the Nsp2 protein of VR-2332. The results showed that the emerging PRRSV, characterized by deletions in Nsp2, is highly pathogenic to pigs. The first outbreaks of highly pathogenic porcine reproductive and respiratory syndrome virus in China occurred in 2006 [9,26]. Compared with the same period in 2006 [9,22], the number of positive cases from Jiangxi Province in 2007 remained unchanged.

## Conclusions

These pig herds were diagnosed as being infected with a highly pathogenic type of North American PRRSV, suggesting it was widespread in Jiangxi Province, China. The results of the present study provide base-line information for further epidemiological survey and control of highly pathogenic PRRSV in China.

## Abbreviations

PRRS: Porcine reproductive and respiratory syndrome; ELISA: Enzyme-linked immunosorbent assay; ORFs: Open reading frames.

## Competing interests

The authors declare that they have no competing interests.

## Authors’ contributions

AG wrote the manuscript; AG, HZ and GW performed sample collection, RNA extraction and RT-PCR; WG and XH performed ELISA of serum; XL participated in sequence analysis; HJ detected classical swine fever virus and porcine circovirus type 2 of the samples; XC and AG were involved in the revision of the manuscript, the project design and the analysis and interpretation of the data assembled in this work. All authors read and approved the final manuscript.
